# Guideline-concordant care and outcomes for pediatric malaria cases: descriptive evidence from pharmacy-based fever management in Kenya

**DOI:** 10.1186/s12936-026-05864-6

**Published:** 2026-04-07

**Authors:** Maria Dieci, Carolyne Nekesa, Nettah Isavwa Kayaro, Jessica Vernon, Ilana Graetz, Kate Winskell, Janet R. Cummings

**Affiliations:** 1 Department of Health Policy and Management, Rollins School of Public Health, Emory University, Atlanta, GA USA; 2 Vyxer Remit Kenya, Busia, Kenya; 3 Maisha Meds, Nairobi, Kenya; 4 Huber Department of Global Health, Rollins School of Public Health, Emory University, Atlanta, GA USA

**Keywords:** Fevers, Pharmacies, Fever management, Febrile illness, Malaria, Children, Kenya, Pediatric, Drug shops

## Abstract

**Background:**

Febrile illness remains a leading cause of morbidity and mortality among children under five in sub-Saharan Africa. In Kenya, pharmacies are a common first point of care for childhood fevers, yet important questions remain about the quality of care provided in these settings and how consistently it aligns with national malaria treatment guidelines. This study examines the care received and outcomes for pediatric suspected malaria illness episodes managed in private pharmacies in malaria-endemic regions of Kenya.

**Methods:**

A longitudinal cohort study was conducted at 39 private stand-alone pharmacies. Caregivers of children under five presenting with fever were surveyed at the point of care and followed up via phone two weeks later. The study assessed whether children received malaria diagnostic testing, whether treatment was guideline-concordant (i.e., antimalarial treatment for confirmed malaria), and whether the child had recovered at follow-up. Logistic regression models using Lasso-selected covariates identified predictors of diagnostic testing and appropriate treatment.

**Results:**

Among 239 febrile children treated at an included pharmacy, 69% received a malaria diagnostic test. Of those tested, 59% were malaria-positive, and 78% of these received appropriate antimalarial treatment. Only 3% of malaria-negative children received antimalarials. Among malaria-positive children treated with antimalarials, 88% had fully recovered at follow-up. Predictors of diagnostic testing included caregiver education, younger child age, and provider malaria knowledge. Appropriate treatment was more likely for older children, male children, and when providers had more years of experience.

**Conclusions:**

Pharmacies in this sample demonstrate high adherence to malaria diagnostic and treatment guidelines for pediatric febrile illness, with strong alignment between diagnosis and treatment and high rates of illness resolution. However, heterogeneity in care quality based on caregiver, child, and provider characteristics suggests opportunities to improve equity and consistency in pharmacy-based care. These findings underscore the importance of pharmacies in malaria case management and highlight the need for further research and policy attention to this critical access point in the healthcare system.

*Trial registration* Not applicable.

**Supplementary Information:**

The online version contains supplementary material available at 10.1186/s12936-026-05864-6.

## Introduction

Febrile illness is a leading cause of death and serious illness for millions of children across sub-Saharan Africa, despite decades-long global efforts in prevention and treatment [1–6]. Despite childhood fevers being highly treatable, many children face barriers in accessing care. A critical, under-studied access point to care for millions around the world is the pharmacy. Pharmacists can administer basic diagnostic tests, dispense medication, and refer patients to other points of care depending on their illness severity, and as such are important brokers of information and health care [7–9]. Pharmacies are crucial access points for care throughout sub-Saharan Africa in large part because of medication availability, proximity to people’s homes and places of work, and shorter wait times than in public dispensaries or clinics [11–16]. In Kenya, a country with a high child mortality rate and disease burden due to febrile illnesses such as malaria, as many as 40–60% of people visit a pharmacy for consultation and/or medication before seeing a doctor or medical provider [7, 8]. Lack of visibility into how childhood fever cases are managed in pharmacy settings is a key barrier towards identifying policy solutions to improve child health outcomes.

Kenya’s national guidelines for the management of malaria emphasize prompt diagnosis and treatment of malaria, including through engagement with the private sector and community-level providers [17]. Despite their importance as first points of access for care, pharmacies typically operate outside the formal health system and are not consistently integrated into national health reporting or regulatory frameworks [18]. Diagnostic testing rates in pharmacies are often low, and antimalarials are frequently dispensed without confirmatory testing because they can be purchased without a prescription [19]. This practice raises concerns about overtreatment and drug resistance, among others [7, 8, 10, 20–22].

Targeted programs, such as malaria test-and-treat initiatives, have sought to improve diagnostic and treatment practices in pharmacies using decision aids, training, and financial incentives. Existing literature evaluating these programs has focused on test and medication purchase. This work found that incentive-based programs targeting testing are effective at encouraging people to test, and many see those increases in testing translate to improved medication targeting, but evidence on this has been more mixed [7, 8, 10, 22]. Despite the promise of programs such as these, challenges remain in ensuring consistent quality of care across pharmacy settings. To address some of these, more evidence is needed on how pharmacies manage pediatric febrile illness and how their practices align with national treatment guidelines. There is also limited research that follows patients over time to assess how pharmacy-based care affects illness resolution, particularly in vulnerable populations such as children under five. This study addresses these gaps by using longitudinal caregiver-reported data to assess the quality of care and outcomes for pediatric malarial febrile illness episodes managed in Kenyan pharmacies.

In this paper, we seek to address these gaps in the literature by examining the care received by pediatric patients with febrile malaria in pharmacy settings, comparing it against clinical guidelines, and analyzing illness episode outcomes in this context. We collected longitudinal data from caregivers of children under five who sought care for their child’s febrile illness at private, stand-alone pharmacies in malaria-endemic counties in Kenya to characterize the quality of care received throughout the illness episode and illness episode outcome. This population is important to study because child fevers are often treated in pharmacy settings in Kenya, and if incorrectly managed, can have serious consequences for child morbidity and mortality [23]. Secondly, we explore caregiver, child, and provider factors associated with guideline-concordant care for suspected malaria in pediatric cases. By understanding guideline concordant care for suspected malaria in children under five who are sick with fever, we contribute to evidence around quality of care received in pharmacies as well as how pharmacy-based care translates into health outcomes for a key population that has broader policy relevance.

## Methods

### Design, sampling, and data collection

This study collected new data at 39 private stand-alone pharmacies in Kenya (see Supplementary Fig. 1 for a map of study sites). Retail pharmacies represent the majority of pharmacies in Kenya, and they are stand-alone locations not affiliated with a hospital which interface directly with patients to conduct basic diagnostic tests and prescribe/dispense medications.

**Fig. 1:**
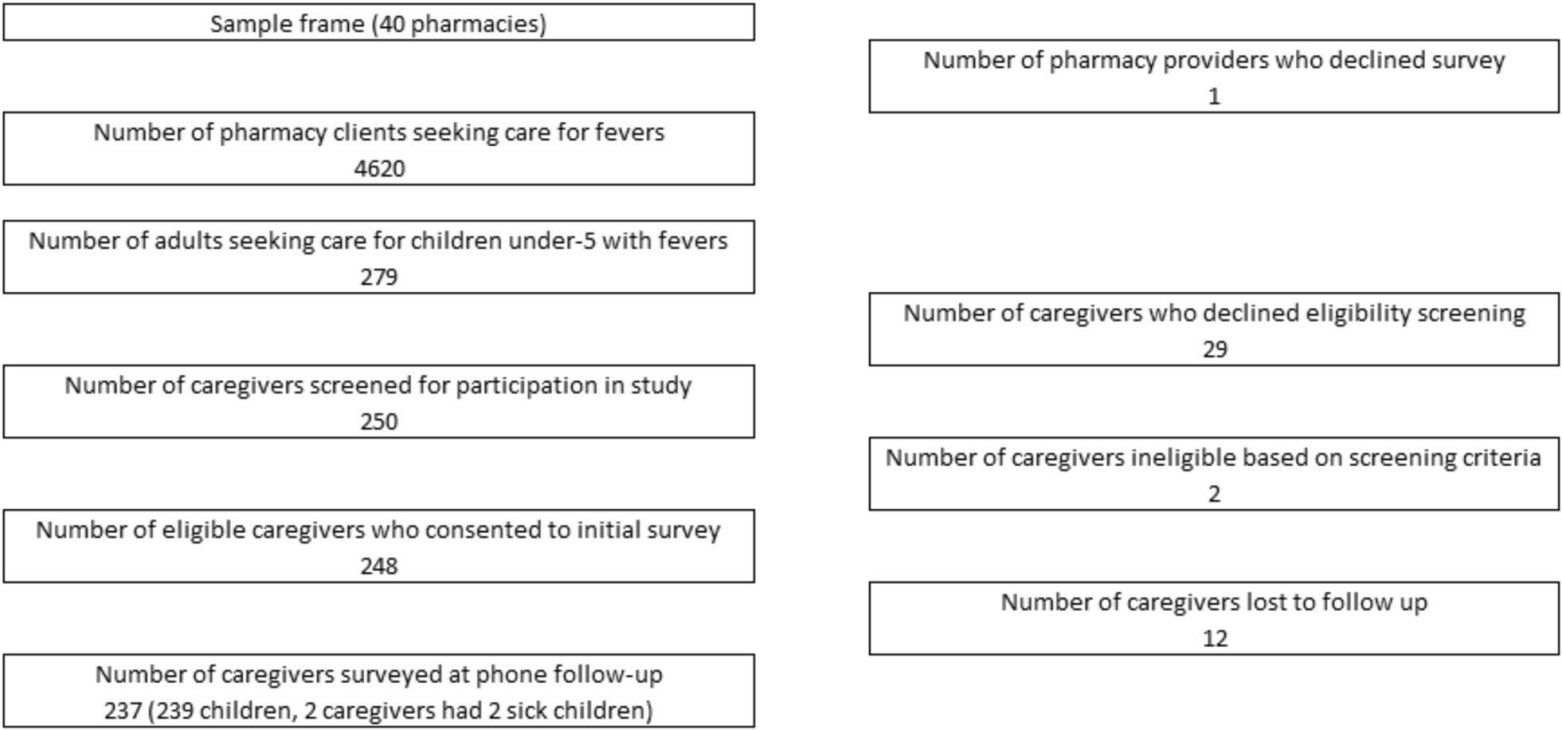
Sample frame and study flow diagram

We used a longitudinal cohort study design, with data collected through an in-person survey at the initial pharmacy visit and a phone survey administered two weeks later. This time period was chosen because most fevers are self-limiting within this time frame [23, 24]. Selection criteria for this study included those (a) who were at least 18 years old, (b) who were primary caregivers of a child under five, (c) who sought care for a child under five who is sick with fever, and (d) who had a working mobile phone and are willing to be contacted for a second phone survey.^1^ Remit Kenya, a third-party survey firm, was responsible for participant recruitment and data collection. In recruiting study participants, a team of 13 enumerators spent three working days (8am-6 pm) at each of 39 pharmacies and invited all eligible caregivers to participate in the study. A total of 250 caregivers were recruited into the study, with 248 meeting all eligibility criteria and consenting to participate. The loss to follow-up between the two survey rounds was 4% (final N = 237). A study flow diagram is in Fig. 1. As an incentive for participation, study participants were given a monetary gift through their mobile phone after completion of both surveys. The survey was conducted between May–June 2024.

### Measures

The main dependent variables for this study can be grouped into two categories: (1) indicators for care received, and (2) illness episode outcomes. Outcomes were measured among survey respondents who participated in both the in-person and phone follow-up surveys for the target child fever illness episode (complete case analysis). For care received, the main dependent variables are whether a symptomatic child received a malaria diagnostic test, and whether medication prescribing to manage child’s illness was guideline-concordant (defined as antimalarial prescribing for malaria-positive cases). For illness episode outcomes, the main dependent variable is whether the child was still sick (symptomatic) at the time of the follow-up phone survey. Fourteen independent variables were tested for association with the outcome variables. These included caregiver and child demographics (caregiver education level, gender, marital status and relationship to household head, child age and gender), provider demographics (provider gender, age, and role at facility), facility location (urban or not), measures of provider knowledge and experience (years worked at facility and percentage of correct malaria symptoms identified). Details on how each of the independent variables was coded can be found in Supplementary Table 1.

### Analysis

**Fig. 2:**
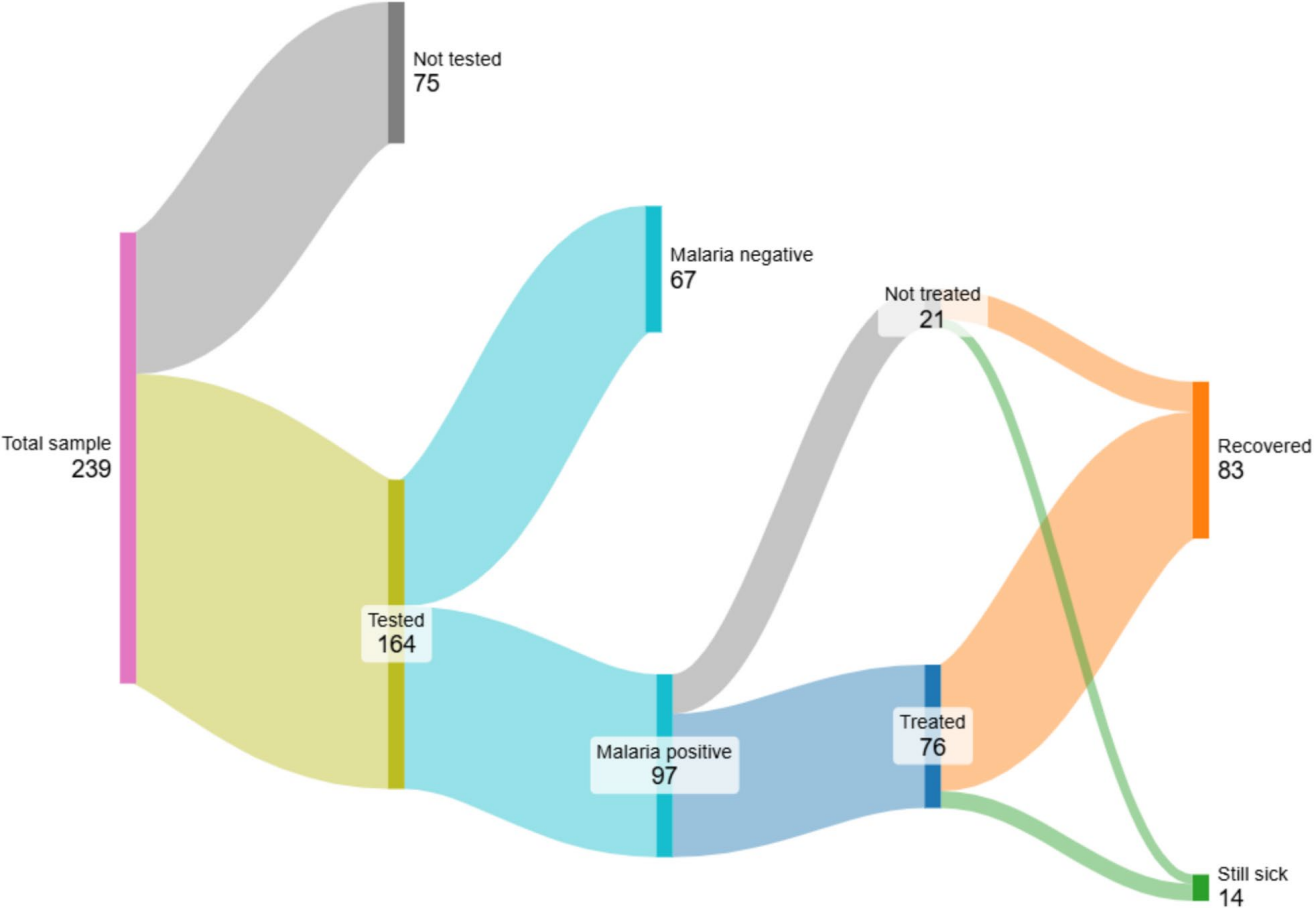
Care and illness episode outcomes for malaria positive pediatric fever cases

First, we describe the sample population (Table 1), reporting percentages and means for each characteristic. We then describe the care and illness outcomes for all children in our sample, by examining diagnostic testing, malaria status, guideline concordant care, and illness episode outcome after two weeks (Fig. 2). We then examine possible predictors of being tested for malaria using the caregiver, child, and provider characteristics discussed above (Table 2). Finally, we examine possible predictors of receiving guideline concordant care for malaria positive cases (Table 3).

**Table 1:** Sample description

	Full sample
	N (%) Mean (SD)
A. Caregiver characteristics (N = 237)	
Caregiver educational attainment	
Less than primary	11 (4.6%)
Primary complete	38 (16.0%)
Some secondary	32 (13.5%)
Secondary complete	156 (65.8%)
Caregiver is female	200 (84.4%)
Caregiver is married	183 (77.2%)
Caregiver is household head	82 (34.6%)
B. Child characteristics (N = 239)	
Female child	130 (54.4%)
Child age	
< 1 year of age	55 (23.0%)
1–2 years of age	74 (31.0%)
2- < 5 years of age	110 (46.0%)
C. Pharmacy/provider characteristics (N = 39)	
Female provider	20 (51.3%)
Provider age	33.026 (6.800)
Provider role	
Attendant	5 (12.8%)
Clinician	21 (53.8%)
Pharmacy technician	5 (12.8%)
Other	6 (15.4%)
Clinician & pharmacy technician	2 (5.1%)
Provider tenure at facility (years)	5.154 (3.158)
Pharmacy located in urban area	17 (43.6%)
Provider malaria knowledge: % of symptoms identified	0.458 (0.189)

**Table 2:** Predictors of malaria testing, symptomatic patients

	(1)	
	Malaria test administered	
	Odds ratio	95% Conf. interval
Education level		
Less than primary school	Ref	Ref
Primary school complete	1.657	[0.404,6.793]
Some secondary school	5.492*	[1.034,29.175]
Secondary school complete	2.883	[0.759,10.948]
Caregiver is married		
No	Ref	Ref
Yes	0.444*	[0.203,0.973]
Caregiver is household head		
No	Ref	Ref
Yes	0.642	[0.322,1.281]
Child age		
< 1 year of age	Ref	Ref
1–2 years of age	1.658	[0.765,3.590]
2- < 5 years of age	6.902**	[2.957,16.108]
Provider is female		
No	Ref	Ref
Yes	0.903	[0.378,2.159]
Provider role		
Attendant	Ref	Ref
Clinician	1.85	[0.693,4.943]
Pharmacy technician	4.164 +	[0.987,17.572]
Other	2.613	[0.650,10.509]
Clinician and pharmacy technician	2.374	[0.606,9.304]
Provider malaria knowledge: % of symptoms identified	4.077 +	[0.831,20.012]
N = 238		

Covariates included in model were selected using a penalized lasso logistic regression of all caregiver and child demographics and provider characteristics. Factors that were retained by that process were included in the final logistic regression. Results are reported as odds ratios

+ p < 0.1; *p < 0.05; ** p < 0.01

**Table 3:** Predictors of malaria treatment, confirmed malaria positive patients

	(1)	
	Purchased antimalarials	
	Odds ratio	95% Conf. interval
Caregiver is household head		
No	Ref	Ref
Yes	2.57	[0.802,8.236]
Female child		
No	Ref	Ref
Yes	0.394	[0.108,1.437]
Child age		
< 1 year of age	Ref	Ref
1–2 years of age	4.213 +	[0.792,22.409]
2- < 5 years of age	10.203*	[1.542,67.493]
Provider role		
Attendant	Ref	Ref
Clinician	5.160 +	[0.750,35.483]
Pharmacy technician	1.733	[0.116,25.916]
Other	1.683	[0.224,12.646]
Clinician & pharmacy technician	0.616	[0.043,8.785]
Provider tenure: number of years worked at facility	1.285	[0.901,1.832]
N = 97		

Covariates included in model were selected using a penalized lasso logistic regression of all caregiver and child demographics and provider characteristics. Factors that were retained by that process were included in the final logistic regression. Results are reported as odds ratios. + p < 0.1; * p < 0.05; ** p < 0.01

Given a long list of potential covariates to include in the models presented in Tables 2 and 3, the risk of overfit in a single model is substantial. We chose not to generate indices or meta-categories for two reasons. First, indices would mask the granularity of data available and cause difficulty in the interpretation of effects for each index variable. Second, indices would likely be of more limited policy relevance, whereas interpretation of specific covariates allows for a more thorough examination of the potential determinants of care trajectory type. We therefore took an agnostic approach to variable selection. We used Stata’s (v.18) Lasso procedure for dimensionality reduction with cross-validation to explore which variables (and associated domains) best explain the variation in malaria diagnostic testing and guideline-concordant treatment for malaria-positives observed in our sample without introducing investigator bias in the selection or consolidation of potential factors affecting these outcomes [25]. We used Stata’s Lasso routine including all 12 categorical and continuous variables, specifying final estimates using tenfold cross-validation. The covariates that were selected during this procedure were then included in the final binary logistic regression models. For the binary indicators of whether a malaria diagnostic test was administered and whether confirmed malaria-positive children received antimalarials to manage their illness, we fit multivariate logistic regressions using Lasso-selected covariates. Results of this exploratory descriptive analysis are reported as odds ratios.

## Results

### Sample characteristics

A total of 237 respondents completed both survey rounds and thus were included in the analysis. This corresponds to data on 239 children with febrile illness, as two caregivers in the sample were caring for two children each. We begin by describing the demographic characteristics of the sample (Table 1). The caregivers surveyed (Panel A) were disproportionately female (84%) and married (77%). Sixty-six percent of respondents had completed (at least) secondary school, 14% had some secondary education, and 16% had completed primary school. In Kenya overall, half the population is female, 48% are married, and 57% have completed (at least) secondary school – so our sample skews more female, married, and more educated compared to the national average [26]. Thirty-five percent of caregivers in the sample identified as the household head, and given the high percentage of women surveyed, this aligns with the national average of 34% of female-headed households [26].

Fifty-four percent of children in the sample (Panel B) were female, 23% were under 1 year of age, 31% were between 1–2, and 46% were between 2–5 years of age. In Kenya, among children under five, 21% are under 1 year of age, 39% are between 1–2 years of age, and 40% are between 2–5 years of age. The age distribution of under-five children in our sample is slightly older than the national average [26].

Individuals in our sample sought care from 39 unique providers, whose characteristics are in Panel C of Table 1. Half of providers were women, with an average age of 33 (SD: 6.8). Most providers (54%) were clinicians (pharmacists, clinical officers, nurses, or doctors), with 13% being pharmacy technicians 13% being attendants, 15% holding another role (lab technicians, health records officers), and 5% holding dual roles. Clinicians have terminal degrees in their specialty field, while pharmacy technicians work under the supervision of pharmacists, and attendants support by performing sales and other clerical/customer-facing duties. Providers had worked at the sample pharmacy for an average of 5.2 years (SD: 3.2), and had moderate knowledge of symptoms of malaria, the most diagnosed febrile illness in this setting, identifying an average of 46% of symptoms correctly in an open-ended survey question. Notably, all providers correctly identified fevers as the most common symptom of malaria.^2^

### Care and illness episode outcomes for pediatric fevers

Figure 2 describes care and illness episode trajectories for all children in our sample, focusing on guideline concordant care to manage malaria. All children in the sample (239) were symptomatic with fever when they sought care in study sites. The majority (164 (69%) from Fig. 2) of children with fever received a malaria diagnostic test at the pharmacy, which is consistent with clinical care guidelines for the diagnosis and treatment of childhood fevers in this setting [6, 17]. Of those that were tested for malaria (164 out of 239 children in the sample), 59% (97 out of 164 tested) were diagnosed with malaria. For those that were not tested for malaria (75 out of 239 children in the sample), 75% did not receive a malaria diagnosis from their pharmacy provider (Supplementary Table 2). This underscores the importance of diagnostic testing in appropriate case management, as diagnosis based on symptoms alone can be incomplete.

We now examine treatment decisions, defined as medication purchase decisions to manage illness, by confirmed malaria status (Fig. 2, and Supplementary Table 3), to document whether medication dispensing is aligned with clinical guidelines. Seventy eight percent of confirmed malaria positive children purchased antimalarials appropriately to manage their illness (76 out of 97 malaria positives), while only 3% confirmed malaria negative individuals purchased antimalarials unnecessarily (2 out of 67 malaria negatives).

Finally, we look at illness status after two weeks for malaria positive cases that had their illness correctly managed with antimalarials. As shown in the figure, 88% of malaria positive children who had their illness managed with antimalarials reported full illness resolution at follow up (67 out of 76 malaria positives that received antimalarials), with an average symptom duration of 5.68 (SD: 3.14) days.

### Predictors of guideline-concordant care for pediatric malaria cases

We look at predictors of diagnostic testing using all covariates from Table 1 and the cross-validation selection option in Stata’s Lasso routine to select lambda with tenfold cross-validation. In this Lasso procedure 7 covariates are selected for inclusion. Including all the categorical variable levels and all continuous variables, this totaled 7 out of 12 possible covariate levels with a lambda of 0.013 (CV mean deviance = 1.19). The chosen set of covariates included three caregiver characteristics, one child characteristic, and three provider characteristics.

Table 2 shows the results of our binary logistic regression with child having received a malaria diagnostic test as the dependent variable and independent variables including only Lasso-selected covariates from the procedure described above. The results presented below should be interpreted as exploratory descriptive findings.

More educated caregivers, older children, and more knowledgeable providers were associated with increased likelihood of diagnostic testing for malaria. Caregivers with some secondary school had 5.49 higher odds than those with less than primary school to have children who were tested for malaria (CI: [1.034, 29.175]). Children who were between 2 and 5 years of age had 6.902 higher odds than those below 1 year of age to be tested for malaria (CI: [2.957, 16.108]) Finally, providers who correctly identified more malaria symptoms had higher odds of testing children under their care for malaria (OR 4.077, CI: [− 0.831, 20.012]).

We now look at predictors of antimalarial dispensing for confirmed malaria positive cases (97 out of 239 cases in our sample) using all covariates from Table 1 and the cross-validation selection option in Stata’s Lasso routine to select lambda with tenfold cross-validation. In this lasso procedure 5 covariates are selected for inclusion. Including all the categorical variable levels and all continuous variables, this totaled 5 out of 12 possible covariate levels with a lambda of 0.04 (CV mean deviance = 1.01). The chosen set of covariates included one caregiver characteristic, two child characteristics, and two provider characteristics.

Table 3 presents this exploratory analysis. Among confirmed malaria positive individuals, older children, male children and more experienced providers were associated with higher odds of antimalarial purchase. Children who are female have lower odds than male children of receiving antimalarials to manage their malaria illness (OR 0.394, CI: [0.108, 1.437]). On average, children between 2 and 5 years of age have 10.203 higher odds than children under 1 year of age of receiving antimalarials to manage their illness (CI:1.542, 67.493]). Clinicians are associated with higher odds of prescribing antimalarials than attendants (OR 5.16, CI: [0.750, 35.483]). And finally, providers with more experience are associated with higher odds of dispensing antimalarials to manage a child’s malaria illness (OR: 1.285, CI: [0.901, 1.832]).

## Discussion

This paper provides novel evidence on how pediatric suspected malaria illnesses are managed in private pharmacy settings and the outcomes of those illnesses. Using longitudinal illness episode data from caregiver surveys and cross-sectional provider survey data, we shed light on an important population and understudied care setting. First, we find high adherence to malaria diagnostic and treatment guidelines for managing suspected malaria for children under five in pharmacies. Second, we find that among children who receive appropriate treatment for confirmed malaria, illness resolution is high. Finally, in exploratory analysis, we find that care quality for child febrile illness management is influenced by caregiver, child and provider characteristics with implications for policy and practice.

Malaria diagnostic and treatment guidelines are adhered to at relatively high rates to manage and treat child fevers in pharmacies. More than two thirds of febrile children received a malaria diagnostic test at the pharmacy where their illness was managed, aligning with clinical care guidelines to test prior to treatment with antimalarials. One possible explanation is that pharmacies in this setting participated in a malaria test-and-treat program that supported guideline-concordant care through decision aids and incentives, as evaluated elsewhere[10]. Diagnostic testing for malaria has historically been limited in pharmacy settings in high malaria burden areas [7,27, 28]. A recent systematic review and RCTs in Kenya found that interventions that promote diagnostic testing through incentives and subsidies lead to higher rates of testing, so our finding is consistent with recent literature [22]. We additionally observe high levels of diagnosis-concordant treatment decisions, consistent with malaria clinical care guidelines. Seventy-eight percent of confirmed malaria-positive children received appropriate antimalarial treatment in pharmacies, while just 3% of malaria-negative children received antimalarials unnecessarily. This contrasts with much of the existing literature, which reports generally high levels of antimalarial prescribing regardless of malaria status in pharmacy settings [14, 20, 29]. Taken together, these findings suggest relatively strong adherence to diagnostic and treatment guidelines in pharmacy-based care in this context.

Effective diagnosis and treatment of malaria-positive cases in pharmacies was associated with high rates of reported illness resolution. Among malaria positive children who received antimalarials at their pharmacy visit, where most child fever cases were managed, 88% reported full illness resolution within two weeks, with an average symptom duration of 5.68 (SD: 3.14) days. While many childhood fevers are self-limiting, malaria requires prompt diagnosis and treatment to avoid prolonged illness and progression into severe forms of the disease, which are particularly dangerous for young children [30]. Given that pharmacies often serve as the first access point to care for common illnesses [15, 16, 31–33], it is crucial to understand care and illness outcomes for cases managed in these settings. Our longitudinal data provide suggestive evidence that appropriate pharmacy-based management of pediatric malaria may be effective in resolving illness.

Finally, we observe that care quality varies across subgroups—with caregiver, child and provider characteristics influencing testing and treatment decisions in different ways. Specifically, more educated caregivers, older children and more knowledgeable providers are more likely to have their pediatric fever case tested prior to treatment. Older, male children and more experienced providers are more likely to have their confirmed pediatric malaria case treated appropriately with antimalarials. More highly educated caregivers may request testing at higher rates, or education could be a proxy for income, with more highly educated caregivers being able to pay the out-of-pocket cost for testing in addition to medication. Age is negatively correlated with the risk of severe illness outcomes for fevers, with children under 1 year of age being most vulnerable [6, 34, 35]. Therefore, it is possible that for providers and caregivers managing fevers in the youngest children, the risks of not initiating treatment immediately outweigh the potential benefit of learning about one’s illness status. Finally, provider knowledge of malaria case management and experience translate directly into better quality practice, a finding which contributes to the literature on the provider know-do gap and points to the potential for training and continued education as a strategy to improve practices [36, 37]. Overall, these findings highlight disparities and opportunities to improve equitable and guideline-concordant care for malaria case management in pharmacy settings.

Our study is not without limitations. First, by sampling caregivers at pharmacies, our study focuses on a pre-selected sample of those who have already made the decision to visit a pharmacy for their child’s illness. As such, we cannot speak to more general patterns in care and illness outcomes, and results must be interpreted with that framework in mind. Second, our sample size of caregivers is small given the pilot nature of this study. Therefore, these results should be seen as descriptive findings that provide suggestive evidence on care and illness episode outcomes for this population. Finally, in this paper we focus on guideline-concordant care for malaria, and do not speak to quality of care and illness episode outcomes for two important groups of cases: children who either were not tested or had non-malarial febrile illnesses. These findings and limitations point to several avenues for future research, which include evidence on care-seeking behavior and quality of care across settings; larger-scale, causal evidence on the role of pharmacy-based programs on fever management; and research on non-malarial febrile illness management and outcomes for pediatric fever cases.

## Conclusions

Our pilot longitudinal dataset provides descriptive evidence on the role of pharmacies in providing care for child febrile illness in Kenya, and highlights an important patient population and an often-overlooked care setting. We observed relatively high adherence to malaria clinical care guidelines for managing child febrile illness in pharmacies. Additionally, our data suggest that malaria-positive children under five who received antimalarials reported relatively quick illness resolution. Finally, we observed that quality of care in pharmacies varied by provider knowledge and caregiver/child characteristics. Taken together, these findings underscore the need for future research in Kenya and other countries where pharmacies represent a large and growing part of the private health care sector, to better understand the care they provide and the populations they serve.

## Supplementary Information

Additional file1

## Data Availability

The data underlying this article will be shared on reasonable request to the corresponding author.

## Footnotes

1 Children under five were selected, given the high burden of febrile illness in this age group and heightened risk of severe illness and adverse outcomes due to febrile illness.

2 Providers were asked to identify symptoms of malaria during the provider survey (without prompting or referring to any lists). Surveyors marked down the ones that providers stated, unprompted, with an option to specify other symptoms if they did not appear in the pre-populated list. Symptoms of malaria (both generalized and severe) that counted as “correct” included fever, headache, chills, malaise/fatigue, difficulty breathing, convulsions, and jaundice, and were generated by the research team in consultation with a clinical consultant and the Kenyan national malaria control guidelines. The proportion correct refers to the number of the above symptoms that the provider identified out of the total (7).
