# Supplementary material for: Guideline-concordant care and outcomes for pediatric malaria cases: descriptive evidence from pharmacy-based fever management in Kenya
Source: Malar J. 2026 Apr 7;25:222. doi: 10.1186/s12936-026-05864-6 (PMC13188472; doi:10.1186/s12936-026-05864-6)
Supplement: Supplementary file 1 — Additional file1 [file 12936_2026_5864_MOESM1_ESM.docx]

**Supplementary Tables and Figures**


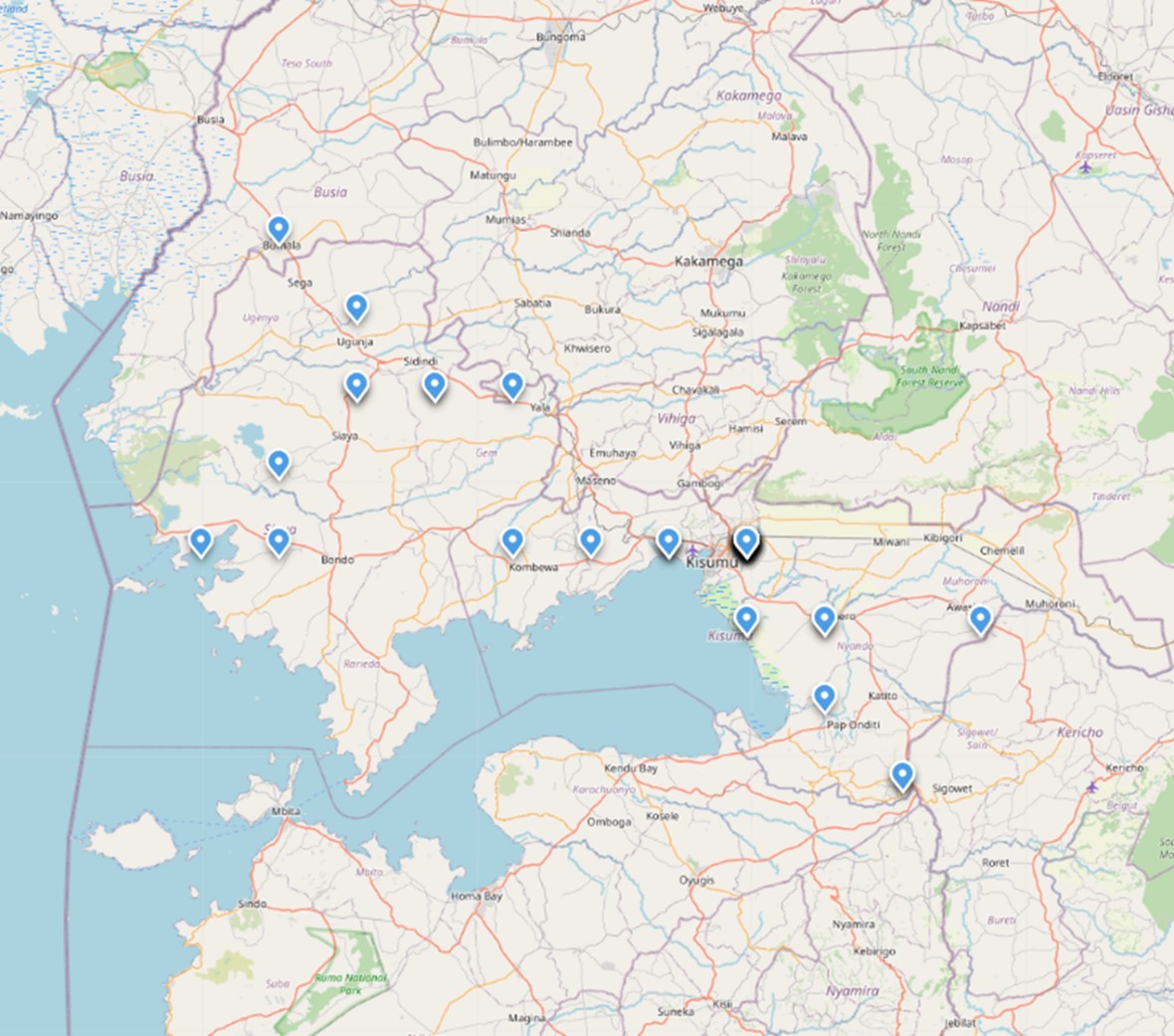


Figure 1. Map of study sites


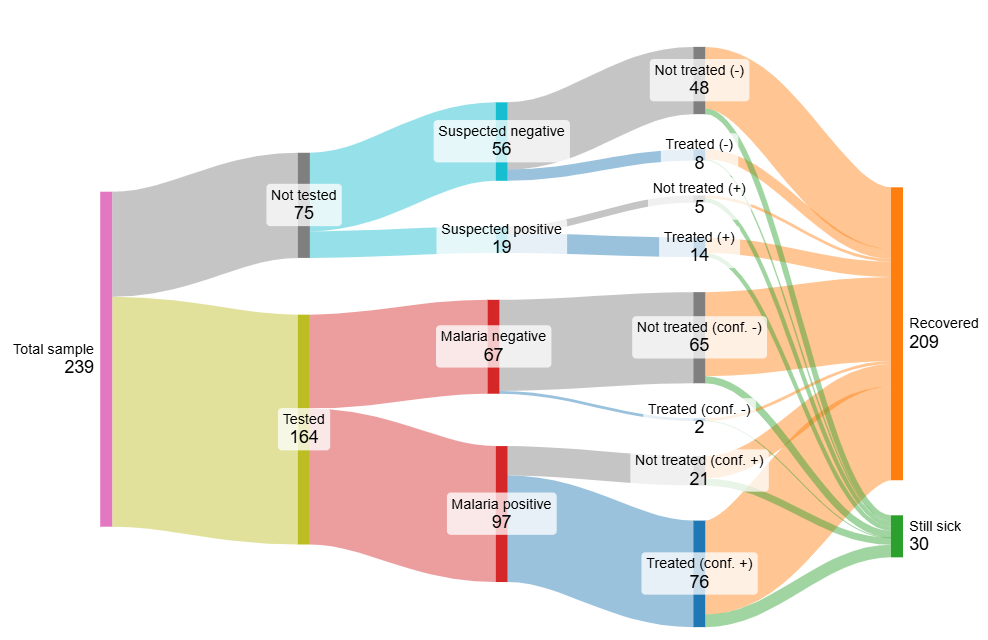


Figure 2. Care and illness episode outcomes for all pediatric fever cases in sample


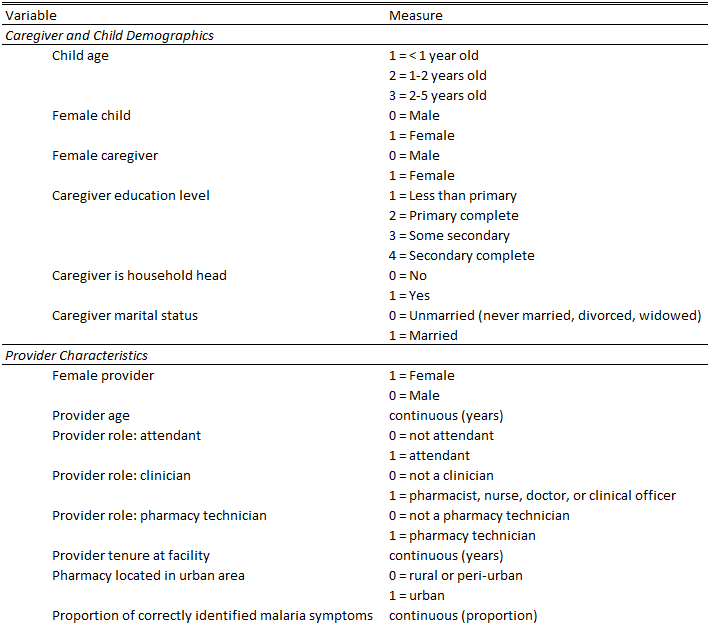


Table 1. Variable descriptions

Table 2. Malaria diagnosis, by testing status

Table 3. Appropriate treatment, by malaria status
